# Lung adenocarcinoma-intrinsic GBE1 signaling inhibits anti-tumor immunity

**DOI:** 10.1186/s12943-019-1027-x

**Published:** 2019-06-20

**Authors:** Lifeng Li, Li Yang, Shiqi Cheng, Zhirui Fan, Zhibo Shen, Wenhua Xue, Yujia Zheng, Feng Li, Dong Wang, Kai Zhang, Jingyao Lian, Dan Wang, Zijia Zhu, Jie Zhao, Yi Zhang

**Affiliations:** 1 Biotherapy Center, The First Affiliated Hospital of Zhengzhou University, Zhengzhou, 450052 Henan China; 2 Department of Pharmacy, The First Affiliated Hospital of Zhengzhou University, Zhengzhou, 450052 Henan China; 3 Cancer Center, The First Affiliated Hospital of Zhengzhou University, Zhengzhou, Henan 450052 People’s Republic of China; 4 Key Laboratory for Tumor Immunology and Biotherapy of Henan Province, Zhengzhou, Henan 450052 People’s Republic of China; 5 Engineering Laboratory for Digital Telemedicine Service, Zhengzhou, Henan 450052 People’s Republic of China; 6 Internet Medical and System Applications of National Engineering Laboratory, Zhengzhou, Henan 450052 People’s Republic of China

**Keywords:** GBE1, STING pathway, Type I interferon, T cell infiltration, PD-L1, Anti-tumor immunity

## Abstract

**Background:**

Changes in glycogen metabolism is an essential feature among the various metabolic adaptations used by cancer cells to adjust to the conditions imposed by the tumor microenvironment. Our previous study showed that glycogen branching enzyme (GBE1) is downstream of the HIF1 pathway in hypoxia-conditioned lung cancer cells. In the present study, we investigated whether GBE1 is involved in the immune regulation of the tumor microenvironment in lung adenocarcinoma (LUAD).

**Methods:**

We used RNA-sequencing analysis and the multiplex assay to determine changes in GBE1 knockdown cells. The role of GBE1 in LUAD was evaluated both in vitro and in vivo.

**Results:**

GBE1 knockdown increased the expression of chemokines CCL5 and CXCL10 in A549 cells. CD8 expression correlated positively with CCL5 and CXCL10 expression in LUAD. The supernatants from the GBE1 knockdown cells increased recruitment of CD8^+^ T lymphocytes. However, the neutralizing antibodies of CCL5 or CXCL10 significantly inhibited cell migration induced by shGBE1 cell supernatants. STING/IFN-I pathway mediated the effect of GBE1 knockdown for CCL5 and CXCL10 upregulation. Moreover, PD-L1 increased significantly in shGBE1 A549 cells compared to those in control cells. Additionally, in LUAD tumor tissues, a negative link between PD-L1 and GBE1 was observed. Lastly, blockade of GBE1 signaling combined with anti-PD-L1 antibody significantly inhibited tumor growth in vivo.

**Conclusions:**

GBE1 blockade promotes the secretion of CCL5 and CXCL10 to recruit CD8^+^ T lymphocytes to the tumor microenvironment via the IFN-I/STING signaling pathway, accompanied by upregulation of PD-L1 in LUAD cells, suggesting that GBE1 could be a promising target for achieving tumor regression through cancer immunotherapy in LUAD.

**Electronic supplementary material:**

The online version of this article (10.1186/s12943-019-1027-x) contains supplementary material, which is available to authorized users.

## Background

In addition to tumor cells, the tumor microenvironment harbors a variety of host-derived cells. It is a complex system playing an important role in tumor development and progression [1]. The tumor microenvironment is associated with many soluble factors and metabolic changes. Within the tumor microenvironment, tumors impose several limitations to dampen T cell immunity. As T cells experience the metabolic framework of growing tumors, they fail to activate distinct pathways necessary to accomplish their function. Moreover, accumulating evidence shows that among the various metabolic adaptations used by cancer cells to adjust to the conditions imposed by the tumor microenvironment, changes in glycogen metabolism is now becoming a prominent feature [2–4]. Our previous study showed that glycogen branching enzyme (GBE1) is downstream of the hypoxia-inducible factor-1 (HIF1) pathway in hypoxia-conditioned lung cancer cells [5] and GBE1 may be a critical regulator in the microenvironment of lung cancer.

Based on previous studies conducted by us and the others, a large number of tumor-infiltrating lymphocytes (TILs) correlates with increased expression of multiple chemokines CCL5 and CXCL10, capable of recruiting effector T cells, by attracting CD8^+^ T lymphocytes into various tumors [6–10]. Understanding the molecular basis of T lymphocyte accumulation in tumors is crucial for the improvement of immune cell-based therapy.

Programmed death ligand-1 (PD-L1), is a critical immune checkpoint molecule exploited by cancer cells to escape immune surveillance [11]. PD-L1 expression on tumor cells and its presence in the tumor microenvironment correlates negatively with the presence of TILs. When PD-L1 is present on the cancer cells, and macrophages bind to programmed cell death protein-1 (PD-1) on activated cytotoxic T lymphocytes (CTLs) at the tumor site, PD-L1-induced inhibitory signal shuts down their anti-tumor activity [12]. However, PD-L1 on tumor cells suppresses the effector function of CD8^+^ T cells [13, 14]. To elucidate whether PD-L1 expression reflects host-tumor immunity, we evaluated CD8^+^ TILs, since the presence of particular TIL subsets correlates with better prognosis in cutaneous melanoma, colorectal cancer, esophageal cancer, renal cancer, and ovarian cancer [15–21].

In the current study, we evaluated the correlation between lung adenocarcinoma (LUAD)-intrinsic GBE1 signaling and anti-tumor immunity, including T cell infiltration and PD-L1 regulation on tumor cells. It appears that knockdown of GBE1 in tumors initiates host immune response via stimulator of interferon genes (STING) pathway and type I interferon (IFN-I) activation. GBE1 may be a negative regulator of the STING pathway, and is envisioned as a part of a negative feedback loop controlling the duration of innate immune activation. Thus, GBE1 may serve as a potential therapeutic target for treating LUAD.

## Methods

### Patients and tumor samples

LUAD tissue samples for immunohistochemistry (IHC) and immunofluorescence analyses were obtained from 30 patients at The First Affiliated Hospital of Zhengzhou University. These patients were subjected to routine laboratory diagnosis, and the samples were analyzed using conventional cytology. Written informed consent was obtained from all patients. The consent procedure was in accordance with the standards defined by the Institutional Review Boards of The First Affiliated Hospital of Zhengzhou University.

### Public clinical datasets and gene set enrichment analysis

We obtained paired RNA-sequencing (RNA-seq) and survival data from 571 LUAD cases in The Cancer Genome Atlas (TCGA) from the Broad Institute’s Firehose (http://xena.ucsc.edu/). Data were downloaded in November 2018 and included RNA-seq data of 571 samples (511 primary LUAD, two recurrent LUAD, and 58 paired non-cancerous (normal) lung tissues). Of the 511 primary LUAD cases, 483 had survival profiles. Therefore, to evaluate the clinical significance of GBE1 in LUAD, we determined the expression of CD8, CCL5, CXCL10, PD-L1, and IFN signature in the 571 LUAD cases from the TCGA dataset. A median expression cutoff value for CD8 and TMEM173 (also called STING) expression was used to analyze overall survival (OS) of the high versus the low expression subgroup, and by the Kaplan-Meier analysis and significance was assessed by a log-rank test. The mean expression of the interferon (IFN)-induced gene set was used to define IFN signature^high^ and IFN signature^low^ subgroups using an unbiased median expression cutoff value. Correlations between PD-L1, IFN signature, CD8, CCL5, and CXCL10 expression were determined using Pearson correlation coefficients. Expression profiles of paired normal lung samples were available for 58 of the 571 cases.

### Cell culture and polyinosinic:polycytidylic acid [poly(I:C)] treatment

The human LUAD cell line A549 was maintained in RMPI 1640 medium (Hyclone, USA) supplemented with 10% fetal bovine serum (FBS, Hyclone, USA), 100 units/ml of penicillin, and 100 μg/ml of streptomycin at 37 °C, in a 5% CO_2_ humidified incubator. Cells were harvested after treatment for 24 h with 100 μg/ml of poly(I:C) (Apexbio, USA).

### Plasmid construction and cell sorting

Small interfering RNAs (siRNAs) against the GBE1 gene were transfected using Lipofectamine 3000 (Life Technologies, USA) at a final concentration of 20 nM following the manufacturer’s instructions. The siRNA sequences of GBE1 were as follows: GBE1 siRNA-1 (target sequence: 5′-GGCAAUCAUGGAGCAUGCUUACUAU-3′), GBE1 siRNA-2 (target sequence: 5′-CCAUGGGUAUCAUAGUCCUCUUAGA-3′), and control siRNA (target sequence: 5′-AAUUCUCCGAACGUGUCACGU-3′). The short hairpin RNA (shRNA) expressing stable A549 cell line was generated by transfecting GBE1 shRNA (shGBE1) into the AgeI/EcoRI site of hU6-MCS-Ubiquitin-EGFP-IRES-puromycin vector (Genechem, China), the following sequence was used: 5′-ACGGAGTCTAAGAATTTAT-3′. Cells infected with lentivirus were sorted by MoFlo XDP (Beckman, USA) based on the expression of green fluorescent protein (GFP). The cells were then harvested and cultured for subsequent functional studies.

### RNA-seq analysis

Total RNA was isolated and used for RNA-seq analysis. cDNA library was constructed and sequenced using the BGISEQ-500 platform (BGI, China). High-quality reads were aligned to the human reference genome (GRCh38) using Bowtie2. The expression levels for each of the genes were normalized to fragments per kilobase of exon model per million mapped reads (FPKM) using RNA-seq by Expectation Maximization (RSEM). The RNA-seq data in this paper have been deposited in the National Center for Biotechnology Information (NCBI) with accession number SUB4719047.

### Multiplex assay

Chemokines can recruit immune-related cells to tumor sites. A multiplex assay was used to detect the levels of immune cell-related chemokines in the cell supernatants derived from cell culture and was analyzed using a multi-analyte flow assay kit (LEGENDplex™, BioLegend, San Diego, USA) according to the manufacturer’s instructions (https://www.biolegend.com/legendplex). This facilitates simultaneous measurement of 13 human chemokines, including CXCL8, CXCL10, CCL11, CCL17, CCL2, CCL5, CCL3, CXCL9, CXCL5, CCL20, CXCL1, CXCL11, and CCL4.

### RNA isolation and quantitative real-time PCR

Total RNA was extracted from lung cancer cells with TRIzol reagent (Invitrogen Life Technologies) according to the manufacturer’s instructions. The concentration and purity of RNA were detected using NanoDrop 2000 (Thermo Scientific). The first-strand cDNA was synthesized from 1 μg of total RNA using PrimeScript RT reagent Kit with gDNA Eraser (TaKaRa). Samples containing 1 μg total RNA were incubated with 1 μl of gDNA Eraser, 2 μl of 5× gDNA eraser buffer and RNase-free dH_2_O at 42 °C for 2 min. Following the addition of the enzyme mix, the reaction was incubated at 37 °C for 15 min. Quantitative real-time PCR was performed using SYBR Premix Ex Taq II (Roche) in Agilent Mx3005P. PCR results were amplified using the following conditions: 40 cycles at 95 °C/30 s, 95 °C/5 s, 60 °C/30 s. The abundance of mRNA for each gene of interest was normalized to β-actin. The data were analyzed by 2^-ΔΔCt^. Details of primer sequences are listed in Table 1.

**Table 1:** The sequences of primers used for quantitative real-time PCR

Gene	Sense Primer (5′ → 3′)	Antisense Primer (5′ → 3′)
β-actin	GCACTCTTCCAGCCTTCCTTCC	TCACCTTCACCGTTCCAGTTTTT
GBE1	GGAGATCGACCCGTACTTGAA	ACATCTGTGGACGCCAAATGA
TMEM173	AGCATTACAACAACCTGCTACG	GTTGGGGTCAGCCATACTCAG
CCL5	CAGTCGTCTTTGTCACCCGA	TGTAACTGCTGCTGTGTGGT
CXCL10	AACTGTACGCTGTACCTGCAT	GCATCGATTTTGCTCCCCTC
PD-L1	GGACAAGCAGTGACCATCAAG	CCCAGAATTACCAAGTGAGTCCT
HLA-A	TGGAGAGGAGCAGAGATACACC	AGAACCAGGCCAGCAATGATG
HLA-B	TCATCTCAGTGGGCTACGTG	GTGTGTTCCGGTCCCAATAC
HLA-C	GGTGGTGCCTTCTGGACAAG	CTCTTCCTCCTACACATCATAGCG
IFI27	TGCTCTCACCTCATCAGCAGT	CACAACTCCTCCAATCACAACT
IFI6	CAGAAGGCGGTATCGCTTTTC	CCTGCATCCTTACCCGCATT
IFNB1	ATGACCAACAAGTGTCTCCTCC	GGAATCCAAGCAAGTTGTAGCTC
IRF7	CCCAGCAGGTAGCATTCCC	GCAGCAGTTCCTCCGTGTAG
MX1	AGCGGGATCGTGACCAGAT	TGACCTTGCCTCTCCACTTATC
OASL	CTGATGCAGGAACTGTATAGCAC	CACAGCGTCTAGCACCTCTT
STAT1	CGGCTGAATTTCGGCACCT	CAGTAACGATGAGAGGACCCT

### Cell isolation and sorting

Peripheral blood mononuclear cells (PBMCs) were isolated within 2 h of sample collection by Ficoll–Hypaque density gradient centrifugation, sequentially using the anti-CD8 MACS magnetic sorting system (Miltenyi Biotec, Germany) according to the manufacturer’s protocol. CD8^+^ T cells were enriched according to the manufacturer’s instructions, and the purity was more than 95%. The purified CD8^+^ T lymphocytes were used in the transwell assay.

### Protein isolation and western blotting analysis

Cells were extracted into cold lysis buffer containing 50 mM Tris-HCl (pH 7.5), 150 mM NaCl, 1 mM EDTA, 1 mM MgCl_2_, 0.5% Triton X-100, phosphatase inhibitor mix (1 mM NaF, 1 mM Na_3_VO_4_, and 1 mM β-glycerol phosphate), and protease inhibitor mix (1 mM PMSF, 2 μg/ml Roche protease inhibitor cocktail (aprotinin, 1 μg/ml leupeptin, and 1 μg/ml pepstatin A). The protein concentration was determined by using the BCA method (Biyuntian, China). The following primary antibodies were used: anti-GBE1 (Abcam, USA), anti-TMEM173 (ProteinTech Group, USA), anti-PD-L1 (Cell Signaling Technology, USA), and β-actin (Santa Cruz Biotech, USA) as control. These primary antibodies were detected with horseradish peroxidase-conjugated anti-IgG, and the detection was performed with the SuperSignal West Femto Maximum Sensitivity Substrate Trial Kit (Pierce, USA). The band images were digitally visualized and quantified with a Fluor Chem FC2 imaging system (Alpha Innotech, USA).

### Flow cytometry analysis

Cells were stained with PE-conjugated anti-PD-L1, PerCp-conjugated anti-7-AAD, APC-Cy7-conjugated anti-CD3, PE-Cy7-conjugated anti-CD8, APC-conjugated anti-IFN-γ, and FITC-conjugated anti-granzyme B antibodies (BioLegend, USA). Dead cells were stained using 7-AAD. Among them, IFN-γ and granzyme B were used for intracellular staining as follows: cells were first fixed with 2% paraformaldehyde and permeabilized with 0.1% saponin in phosphate buffered saline (PBS) buffer. Next, cells were incubated in the dark for 15 min on ice with antibodies labeled with fluorochrome. For surface assessment, cells were incubated with fluorochrome-labeled antibodies directly. The cell phenotype was determined using cytofluorimetric analysis by flow cytometer (BD FACSCanto II, USA).

### IHC and immunofluorescence staining

The protocols used for IHC and immunofluorescence were performed according to previous studies [22]. Anti-GBE1 (1:300; Abcam, USA), anti-PD-L1 (1:300; Cell Signaling Technology, USA), CD8 (1:300; Abcam, USA), CCL5 (1:300; BioLegend, USA), CXCL10 (1:300; Ruiyingbio, China) were used as primary antibodies. For IHC, three fields of view per sample were imaged. The intensity of immunostaining was taken into consideration when analyzing the data. The percentage scoring of immunoreactive tumor cells was as follows: 0 (< 10%), 1 (10–40%), 2 (40–70%), and 3 (> 70%). The staining intensity was visually scored and stratified as follows: 0 (negative), 1 (yellowish), 2 (light brown), 3 (dark brown). The intensity of staining was obtained by multiplying the two items into a total score, and the scores ranged from 0 to 9. In immunofluorescence, Cy3- and FITC-conjugated secondary antibodies (BioLegend, California, USA, 1:500) were used to detect the primary antibodies. Nuclear staining was performed with DAPI (1:1000; Roche, USA). The samples were visualized with a fluorescence microscope (Olympus, IX71, Japan).

### Xenograft model in nude mice

To generate a subcutaneous xenograft mouse model, 20 female NOD-SCID (NSG) immunodeficient mice (Vital River Laboratory Animal Technology Co. Ltd., China) aged 4–6 weeks, weighing 16–20 g, were divided randomly into four groups (5 mice/group). For establishing the lung cancer xenograft model, the four groups received hypodermic injections of either scrambled shNC or shGBE1 A549 cells (5 × 10^6^ cells in 100 μl PBS) (day-33). Two weeks following hypodermic cell implantation, tumor volumes were measured and calculated using the following formula: (length × width^2^)/2. Mice were treated with or without anti-PD-L1 antibody (1 mg/ml; Biolegend, USA) three times per week for a total of 2 weeks from day-14 to day-1. On day 0, the mice were injected with 1 × 10^6^ human allogeneic PBMCs in the tail vein. Three days later (d + 3), mice were anesthetized with 10% chloral hydrate and sacrificed by cervical dislocation. The tumors were collected to analyze the frequency and function of TILs through flow cytometry and IHC. All mice were housed and maintained under specific pathogen-free conditions. All animal experiments were conducted following the Guide for the Care and Use of Laboratory Animals and were approved by the Institutional Animal Care and Use Committee of the First Affiliated Hospital of Zhengzhou University (No.11400700323829).

### Statistical analysis

Data analysis was performed using SPSS 19.0 statistical software or Prism 6 (Graph Pad Software Inc.). Based on the distribution level, data were expressed as the mean ± standard deviation (SD). Independent-Sample or paired t-test was performed to analyze the differences between two groups with normally distributed continuous variables. Pearson’s coefficient correlation or linear regression analysis was used to analyze the relationships between the expression levels of specific genes. The chi-square test was performed to quantify the IHC correlation of patient-derived samples. The Kaplan-Meier method was used to establish survival curves, and the survival differences were compared using the log-rank test. In all cases, a two-tailed *P*-value < 0.05 was considered statistically significant, *p < 0.05, ** *p* < 0.01, ***p < 0.001.

## Results

### GBE1 prevents CCL5 and CXCL10 expression in LUAD cells

**Fig. 1:**
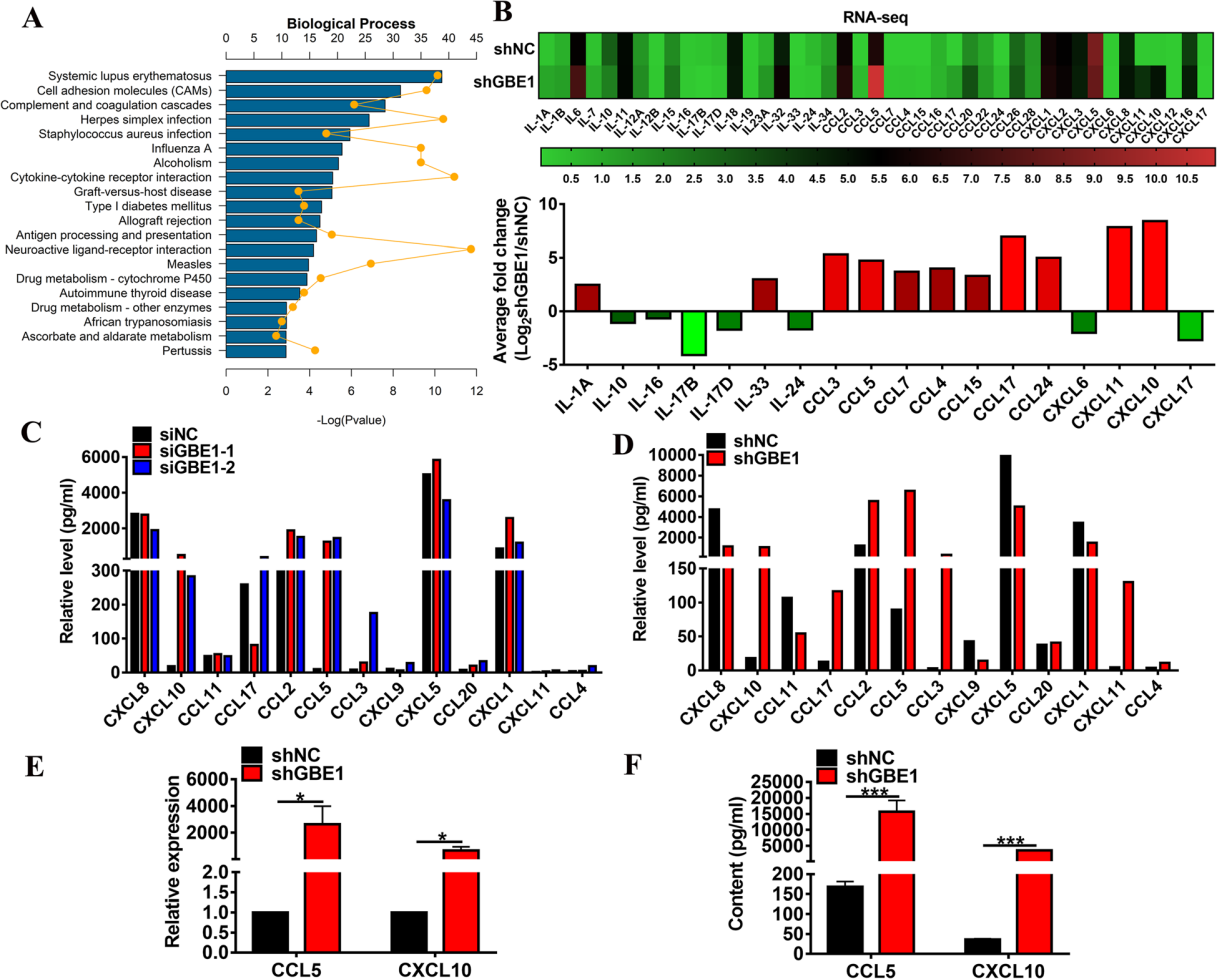
GBE1 prevents CCL5 and CXCL10 expression in LUAD cells**. a** GO analysis showed the top 20 genes involved in shGBE1 A549 cells compared to control based on the RNA-seq data. **b** Heatmap of relative mRNA expression for cytokine-cytokine receptor interaction related genes in shGBE1 A549 cells compared to control. Concentrations of cytokine-cytokine receptor interaction related core-enriched genes in siGBE1 **(c)** and **(d)** shGBE1 A549 cells compared to control analyzed using a multiplex assay (siGBE1: 2 × 10^4^ cells/well, shGBE1: 5 × 10^4^ cells/well). **e** Real-time PCR and **(f)** ELISA analysis of CCL5 and CXCL10 expression in shGBE1 A549 cells compared to control

Increasing evidence indicates that changes in glycogen metabolism of cancer cells are emerging in response to the tumor microenvironment [2]. Our previous study showed that GBE1 is downstream of the HIF1 pathway in hypoxia-conditioned lung cancer cells [5]. To identify the association between the GBE1 pathway and the regulation of LUAD microenvironment, we analyzed the RNA-seq data of shGBE1 and the control A549 cells by using Gene Ontology (GO) analysis. The results showed that a significant difference in expression was related to cytokine-cytokine receptor interactions (Fig. 1a). Furthermore, data based on the RNA-seq analysis revealed that the knockdown of GBE1 in A549 cells significantly upregulated or downregulated the expression of cancer immune-related cytokines and chemokines (Fig. 1b). To support the abovementioned RNA-seq findings, a multiplex assay was performed to evaluate the expression levels of immune cell-related chemokines in siRNA-mediated (siGBE1) and shRNA-mediated knockdown of GBE1 (shGBE1) in comparison with the control. The results showed an increase in the expression levels of CCL5 and CXCL10 in siGBE1 and shGBE1 A549 cells compared to control (Fig. 1c, d). To further validate the inhibitory effect of GBE1 on the production of chemokines CCL5 and CXCL10, we applied real-time PCR and ELISA to analyze the expression levels of CCL5 and CXCL10 in shGBE1 and control cells. Knockdown of GBE1 increased CCL5 and CXCL10 expression in A549 cells (Fig. 1e, f). Additionally, GBE1 overexpression decreased the secretion of CCL5 and CXCL10 in A549 cells (Additional file 1: Figure S1). These data indicate the requirement of GBE1 for the production of CCL5 and CXCL10 in A549 cells. Collectively, GBE1 prevents CCL5 and CXCL10 secretion in LUAD cells, which may further affect the recruitment of T lymphocytes into the tumor microenvironment.

### Knockdown of GBE1 promotes CD8^+^ T cell infiltration mediated by increased CCL5 and CXCL10

**Fig. 2:**
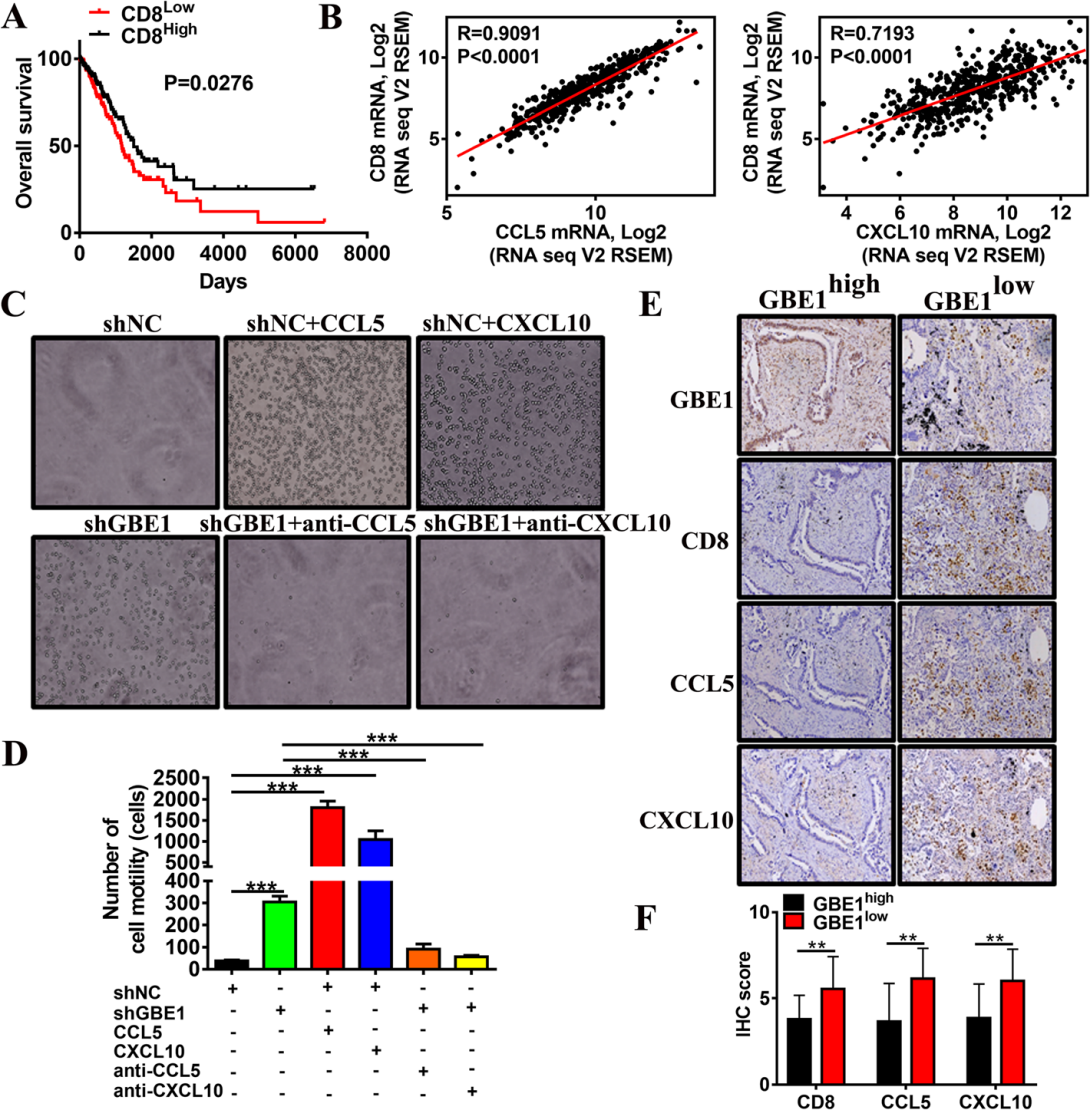
Knockdown of GBE1 promotes CD8^+^ T cell infiltration mediated by increased CCL5 and CXCL10. **a** Kaplan-Meier analysis of OS (calculated as years to death or years to last follow-up) using the TCGA cohort median expression value cutoffs for CD8. *P*-value was determined by a log-rank test. **b** The correlation analysis of TCGA RNA-seq data for CD8 with CCL5 or CXCL10 expression across primary LUAD samples. Pearson correlation coefficients (r) are indicated. Expression values represent log_2_-transformed normalized RNA-seq readings generated with the Illumina platform. **(C)** Transwell assay analysis of CD8^+^ T cell migration ability in the tumor supernatants of shGBE1 A549 cells with treatment of CCL5 or CXCL10 antibodies, or shNC cells with the recombinant protein of CCL5 or CXCL10. **d** Quantification numbers of CD8^+^ T cells passed through the Matrigel matrix by the indicated conditions. **e** IHC analyses of serial sections derived from patients with LUAD (*n* = 30) were stained for GBE1, CD8, CCL5, CXCL10, and divided into two parts according to GBE1 high (left panel) and low expression (right panel). **f** IHC score of CD8, CCL5, and CXCL10 in tumor tissues from LUAD patients analyzed by IHC

Since both CCL5 and CXCL10 attract CD8^+^ T cells into tumor sites [6–10], we next analyzed the effect of CD8 on the survival of patients from TCGA datasets. Subjects with high CD8 expression had greater survival than those with low CD8 expression (Fig. 2a), consistent with the notion that preexisting antitumoral immune responses determine a favorable prognosis in patients with LUAD. Furthermore, CD8 expression was positively correlated with the secretion of CCL5 and CXCL10 in LUAD (Fig. 2b), indicating that local production of CCL5 and CXCL10 may attract CD8^+^ T lymphocytes to tumor sites. Similar results were reported in our previous study on esophageal squamous cell carcinoma [10]. Using transwell assays, we demonstrated that the supernatants derived from GBE1 knockdown cells or those treated with recombinant human CCL5 and CXCL10 robustly enhanced the recruitment of CD8^+^ T lymphocytes; however, the neutralizing antibodies for CCL5 or CXCL10 significantly hampered the cell migration induced by shGBE1 cell supernatants (Fig. 2c, d). These findings were validated by immunohistochemical serial sections, which indicated a negative correlation between GBE1 and CD8^+^ T lymphocyte infiltration in tumor tissues. Tissues with a high expression of GBE1 (score = 2, 3) showed a decrease in CD8 expression, as well as CCL5 and CXCL10 staining (Fig. 2e, f). In contrast, tissues with a low score of GBE1 (score = 0, 1) showed a higher level of CD8 expression (Fig. 2e, f). Accordingly, we propose that GBE1 reduces the recruitment of CD8^+^ T cells into the tumor microenvironment by inactivating CCL5 and CXCL10, thus contributing to the immune escape in LUAD.

### Requirement of IFN-I pathway for the effect of GBE1 knockdown on CCL5 and CXCL10 upregulation

**Fig. 3:**
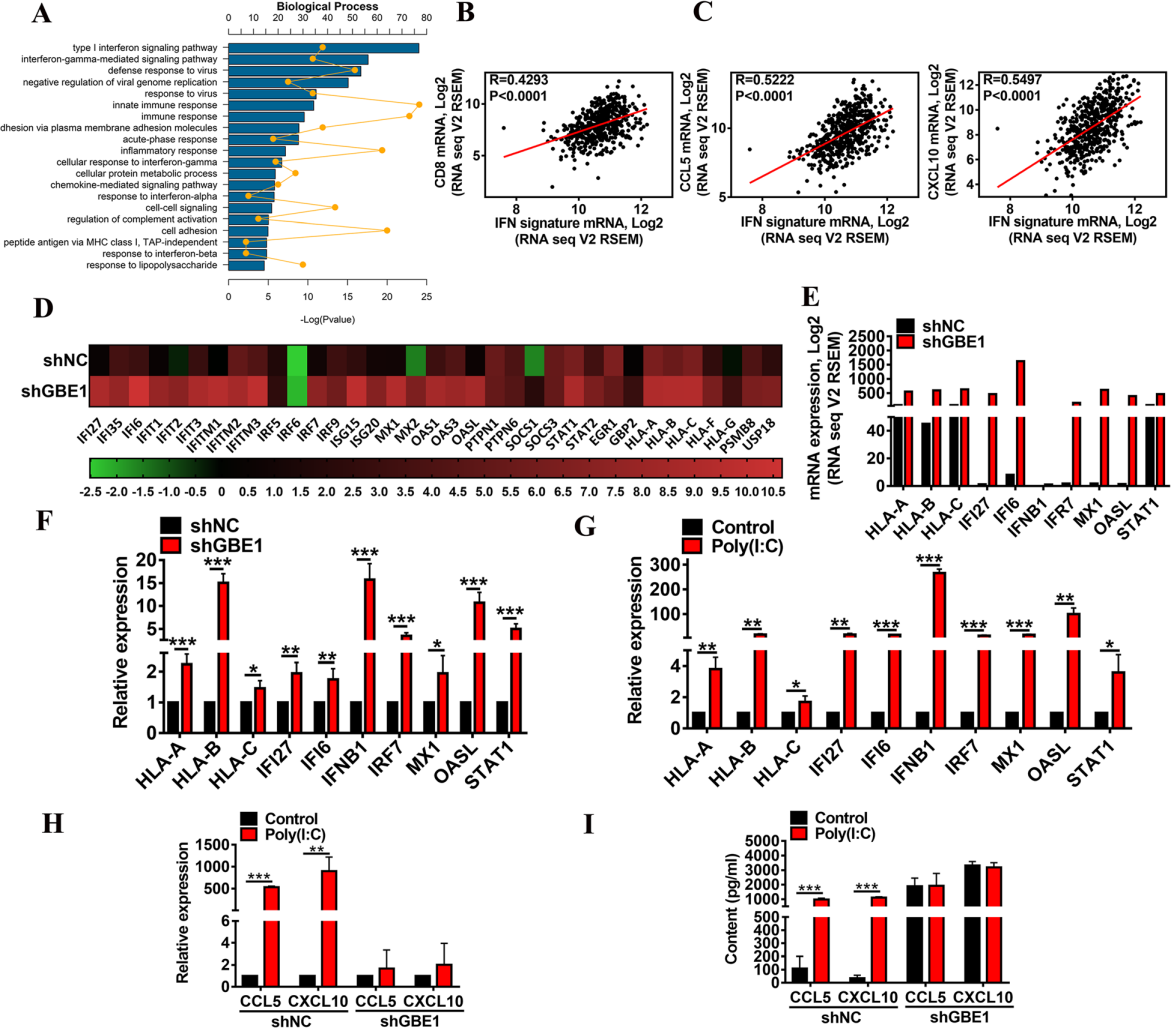
IFN-I pathway is required for the effect of GBE1 knockdown on CCL5 and CXCL10 upregulation. **a** DAVID analysis of the top 20 altered pathways using KEGG in shGBE1 A549 cells compared to control. The correlation analysis of TCGA data for IFN response signature and CD8 **(b)** or CCL5, CXCL10 **(c)** expression. **d** Heatmap of relative mRNA expression for IFNα/β-signaling pathway core-enriched genes in shGBE1 A549 cells compared to control. **e** Quantification of IFNα/β pathway core-enriched genes differentially expressed in shGBE1 A549 cells compared to control. **f** The real-time PCR analysis of mRNA expression of IFNα/β pathway in shGBE1 A549 and control cells. **g** The real-time PCR analysis of the core-enriched genes differentially expressed by the indicated poly(I:C) in A549 cells. Real-time PCR **(h)** and ELISA **(i)** analysis of CCL5 and CXCL10 before and after treatment with poly(I:C) in shNC and shGBE1 A549 cells

To evaluate which signaling pathway mediated the knockdown effect of GBE1 for CCL5 and CXCL10 upregulation, we analyzed the data based on the RNA-seq between shGBE1 and control cells using Kyoto Encyclopedia of Genes and Genomes (KEGG) pathway analysis. The result showed that the type I interferon signaling pathway was the most prominent of the top 20 altered pathways (Fig. 3a). Clinical follow-up data from the TCGA LUAD project showed a strong correlation between IFN-responsive gene expression and CD8 (Fig. 3b), as well as CCL5 and CXCL10 secretion (Fig. 3c). Moreover, we observed that knockdown of GBE1 effectively induced IFNα/β pathway-related genes (Fig. 3d, e), and the findings were validated with real-time PCR analysis (Fig. 3f). As an experimental strategy, we used poly(I:C), which triggers the innate viral recognition receptors TLR3 and MDA5 and efficiently stimulates the IFN-I system [23]. Poly(I:C) treated A549 cells strongly induced the expression of IFN-I-regulated genes (Fig. 3g). Importantly, poly(I:C) also promoted the production and secretion of CCL5 and CXCL10 in A549 cells in vitro. However, CCL5 and CXCL10 secretion was not significantly higher in poly(I:C) treated shGBE1 A549 cells than in untreated shGBE1 cells (Fig. 3h, i). The above data suggest that IFN-I pathway is essential for CCL5 and CXCL10 secretion induced by GBE1 knockdown in LUAD.

### Involvement of STING signaling for CCL5 and CXCL10 production after GBE1 knockdown

**Fig. 4:**
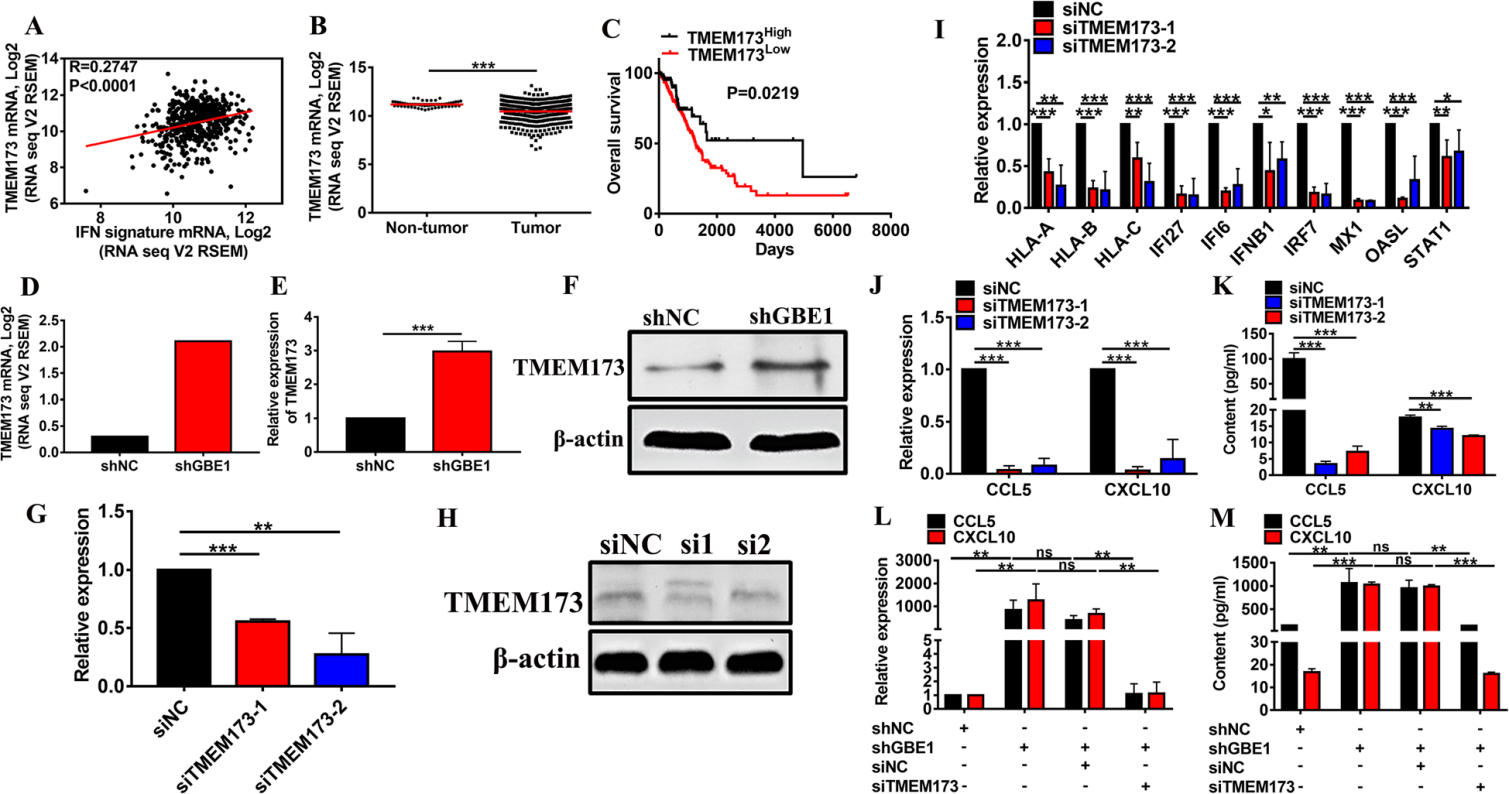
Involvement of STING signaling in CCL5 and CXCL10 expression after GBE1 knockdown. **a** The correlation analysis of TCGA data for IFN response signature with TMEM173 expression across primary LUAD samples. **b** The box plots of TMEM173 expression in normal lung and tumor tissues from TCGA dataset. **c** Kaplan-Meier analysis of OS using the TCGA cohort median expression value cutoffs for TMEM173. The RNA-seq **(d)**, mRNA **(e)** and protein **(f)** expression of TMEM173 in shGBE1 A549 cells compared to control. Real-time PCR **(G)** and western blotting **(h)** analysis of TMEM173 knockdown by siRNA. **i** The real-time PCR analysis of IFNα/β pathway expression with or without TMEM173 knockdown. Real-time PCR **(j)** and ELISA **(k)** analysis of CCL5 and CXCL10 expression with or without TMEM173 knockdown. Real-time PCR **(l)** and ELISA **(m)** analysis of CCL5 and CXCL10 in shGBE1 A549 cells compared to shNC cells with or without TMEM173 knockdown

STING plays an important role in innate immunity. STING induces IFN-I production when cells are infected with intracellular pathogens [24]. We investigated whether STING signaling is associated with CCL5 and CXCL10 secretion after GBE1 knockdown. The expression of TMEM173 significantly correlated with the expression of IFN-I-responsive genes in LUAD tissues from TCGA dataset (Fig. 4a). Clinical follow-up data from the TCGA LUAD cohort revealed that the expression of TMEM173 was significantly lower in tumor tissues than in paired normal lung tissues (Fig. 4b). Subsequently, we classified samples by unbiased median expression value cutoffs and found that high expression of TMEM173 was associated with a favorable disease course (Fig. 4c). The activation of the interferon signaling pathway from GBE1 knockdown was linked with STING signaling. Moreover, mRNA and protein expressions of TMEM173 were dramatically increased in shGBE1 A549 cells (Fig. 4d-f). To further evaluate the effect of STING on CCL5 and CXCL10 production mediated by IFN-I, we utilized two RNA sequences, each directed against a different site of the TMEM173 transcript. The knockdown of TMEM173 was confirmed by real-time PCR and western blotting (Fig. 4g, h). siRNA-mediated knockdown of TMEM173 inhibited the expression of IFNα/β pathway-related genes, including antigen presentation (Fig. 4i). Furthermore, the inhibition of TMEM173 decreased the production and secretion of CCL5 and CXCL10, showing the requirement of STING signaling for chemokine production in A549 cells (Fig. 4j, k). Notably, we observed that GBE1 knockdown, upregulated the production and secretion of CCL5 and CXCL10, while the simultaneous inhibition of TMEM173 demonstrated no significant induction in both genes, reinforcing the importance of STING signaling in chemokine production (Fig. 4l, m). These data support that the augmentation of IFNα/β pathway by GBE1 knockdown partially relies on activating STING signaling, and STING signaling is involved in CCL5 and CXCL10 production in response to GBE1 knockdown.

### The effect of GBE1 on PD-L1 expression

**Fig. 5:**
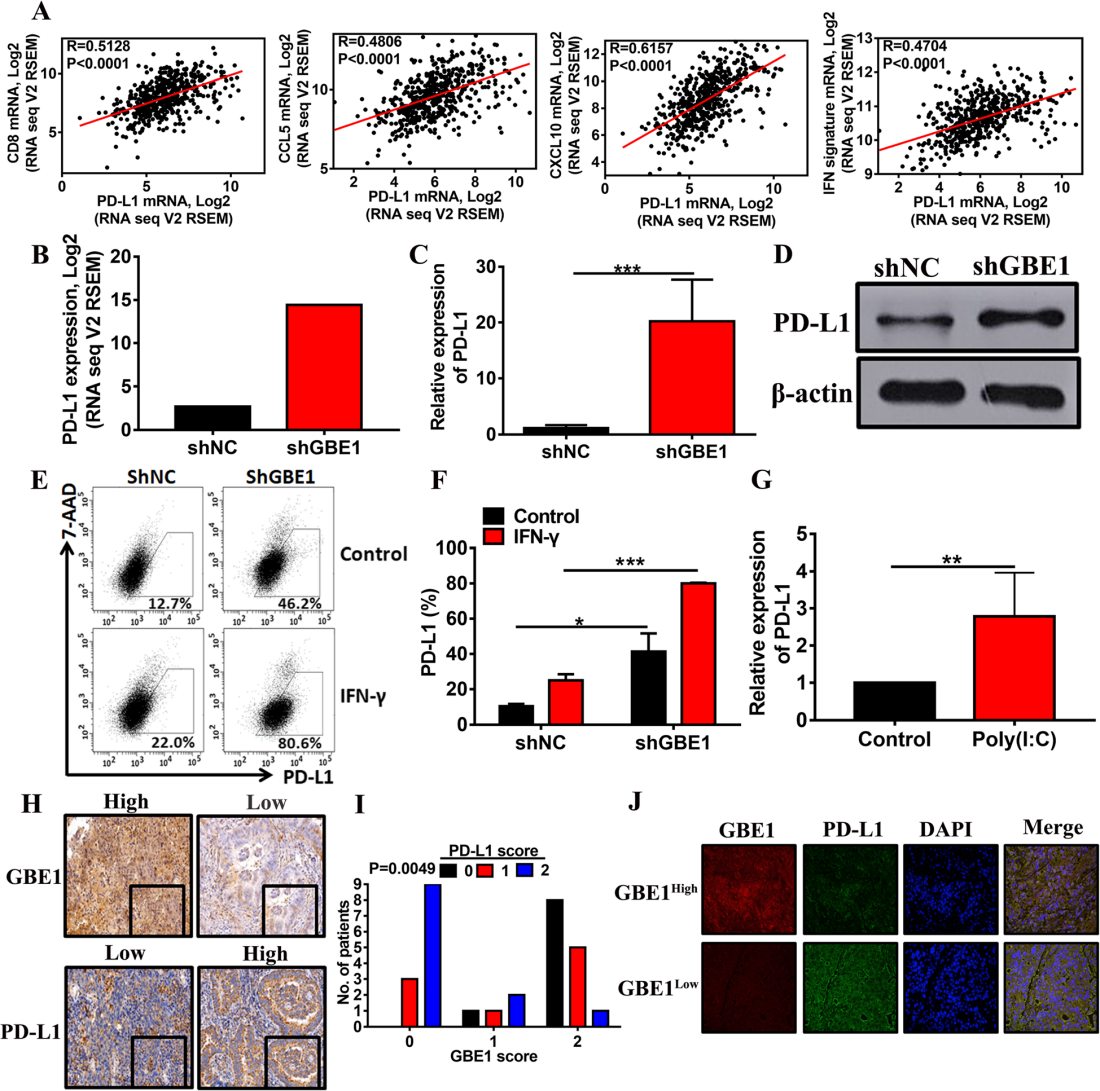
The effect of GBE1 on PD-L1 expression. **a** The correlation analysis of TCGA data for PD-L1 with CD8, CCL5, CXCL10, and IFN response signature expression across primary LUAD samples. The RNA-seq **(b),** mRNA **(c)** and protein **(d)** expression of PD-L1 in shGBE1 A549 cells compared to control. **e and f** Flow cytometry analysis of PD-L1 in shGBE1 A549 cells compared to control with or without IFN-γ treatment. **g** The real-time PCR analysis of PD-L1 in A549 cells treated with poly(I:C). **h and i** IHC interaction plots of serial sections derived from patients with LUAD (n = 30) were stained for GBE1 and PD-L1. **j** Immunofluorescence analysis of GBE1 and PD-L1 expression in LUAD tissues

PD-L1 expression on antigen presenting cells is initially induced in response to IFN-I [25], and this upregulation leads to CD8^+^ T cell exhaustion. So, we hypothesized that GBE1 affects PD-L1 expression on LUAD cells. We showed that, in tumor lesions, the local expression of PD-L1 positively associates with the expression of CD8, CCL5, CXCL10, and also IFN response signature from the TCGA datasets (Fig. 5a). The RNA-seq, mRNA, and protein expression of PD-L1 were significantly increased in shGBE1 A549 cells compared to those in control cells (Fig. 5b-d). A previous study has established that PD-L1 expression in most tumors is induced initially in response to IFN-γ secreted by CD8^+^ T cells recruited to the tumor site as part of an adaptive tumor resistance [26]. In the present study, we observed an induction of PD-L1 mediated by GBE1 knockdown, which was further augmented by IFN-γ induction (Fig. 5e, f). Since poly(I:C) is a potent inducer of IFN-I [27], we assumed and validated that treatment with poly(I:C) could significantly augment the expression of PD-L1 on LUAD cells in vitro (Fig. 5g). Furthermore, we examined the relationship between GBE1 and PD-L1 expression in human LUAD tissues by IHC, using a tissue microarray of 30 LUAD cases. Tissues with a higher score for GBE1 (score = 2) showed decreased PD-L1 expression. In contrast, tissues with a lower score for GBE1 (score = 0, 1) showed a higher level of PD-L1 expression (Fig. 5h, i). PD-L1 protein expression was elevated in low-GBE1 expression areas of the tumor tissues and vice versa, as determined by immunofluorescence assay (Fig. 5j). All these results indicate a negative correlation between PD-L1 and GBE1 in LUAD.

### Blockade of GBE1 signaling combined with anti-PD-L1 antibody inhibits tumor growth in vivo

**Fig. 6:**
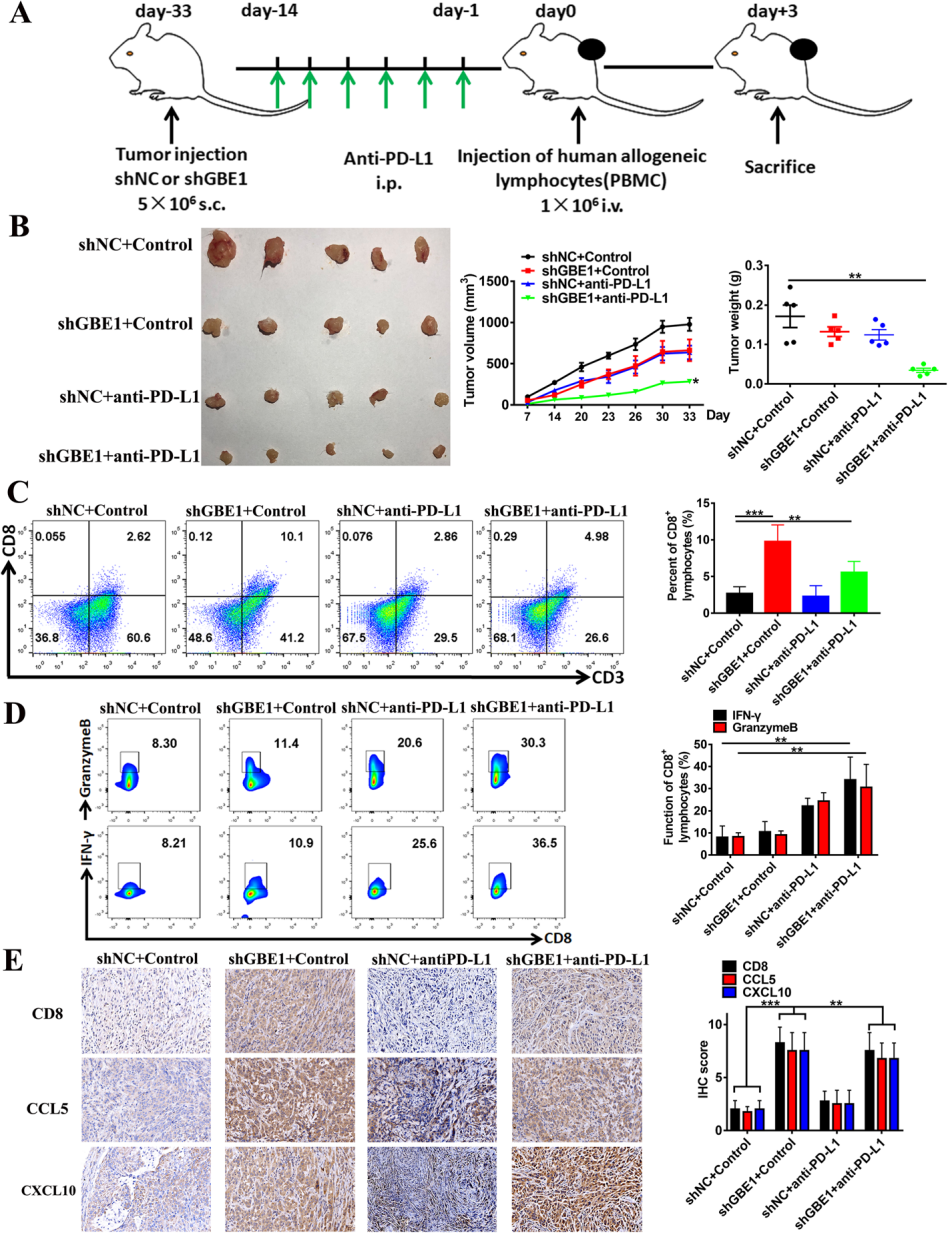
Blockade of GBE1 signaling combined with anti-PD-L1 antibody inhibits tumor growth in vivo. **a** Scheme of the in vivo experimental procedure detailed in “Materials and Methods” and “Results” section. **b** Tumor growth was measured twice a week until mice were sacrificed (*n* = 20). Tumor volumes were measured from day 14 to day 33 after cell implantation. Tumor weights were measured at day 33 after xenograft collection. Flow cytometry analysis of CD8^+^CD3^+^ T cell frequency **(c)** and IFN-γ^+^CD8^+^ or Granzyme B^+^CD8^+^ T cell frequency **(d)** in the tumor xenografts. **e** Illustration of CD8, CCL5 and CXCL10 IHC staining of sections from one representative xenograft

Our experimental results confirm the hypothesis that GBE1 inhibits T cell infiltration in the tumor microenvironment and increases PD-L1 expression in tumor cells. Upregulation of the PD-1/PD-L1 signaling axis in tumor tissues, as a consequence of IFN-I activation and invasion by T cells, predicts therapeutic benefit from PD-L1/PD-1 blockade alone. Thus, blockade of GBE1 could increase the efficacy of immune checkpoint therapy. Next, we tested whether blockade of GBE1 combined with anti-PD-L1 antibody increases anti-tumor immunity to cause sustained inhibition of tumor growth in vivo. We injected shGBE1 and shNC A549 cells into immunodeficient NSG mice [28]. Tumors were treated with or without anti-PD-L1 antibody (1 mg/ml) three times per week for a total of 2 weeks from day-14 to day-1. At day 0, the mice were injected with 1 × 10^6^ human PBMCs. Three days later (d + 3), the mice were sacrificed, and the tumors collected for analysis (Fig. 6a).

The tumor growth, volume, and weight in mice injected with shGBE1 A549 cells and allogeneic lymphocytes were controlled partially compared to the control. This phenomenon may possibly be due to GBE1 knockdown, which suppressed tumor development by non-immune pathways leading to the upregulation of PD-L1 expression on A549 cells, and simultaneously reduced tumor growth at a rate similar to that seen in the group that received anti-PD-L1 alone (Fig. 6b). In addition, we also administered anti-PD-L1 antibody into shGBE1 tumor-bearing mice. In this setting, anti-PD-L1 antibody combined with GBE1 blockade remarkably delayed tumor growth (Fig. 6b). Using flow cytometry analysis, we observed a higher infiltration of CD8^+^ T cells and higher production of T cell function-related molecules IFN-γ, Granzyme B from CD8^+^ T cells in the xenografts from mice treated with shGBE1 A549 cells combined with anti-PD-L1 antibody therapy compared to control groups (Fig. 6c, d). This was indeed confirmed in the xenografts by IHC analysis, which stained tumor sections for CD8, CCL5, and CXCL10 (Fig. 6e). Although CCL5 can also promote the recruitment of regulatory T cells (Tregs) into the tumor site, we did not find significant differences in Treg infiltration (FOXP3 expression) in the xenograft tissues between the four groups by IHC (Additional file 2: Figure S2). In addition, our current study suggests that the application of PD-L1 blockade can inhibit the secretion of tumor CCL5. This partially explains how the combination of anti-PD-L1 with GBE1 knockdown slightly reduced CD8^+^ T cell infiltration compared to GBE1 knockout alone. Therefore, blockade of GBE1 combined with anti-PD-L1 antibody can serve as a potential therapeutic strategy for LUAD.

### Bioinformatics prediction and full-text mode diagram

**Fig. 7:**
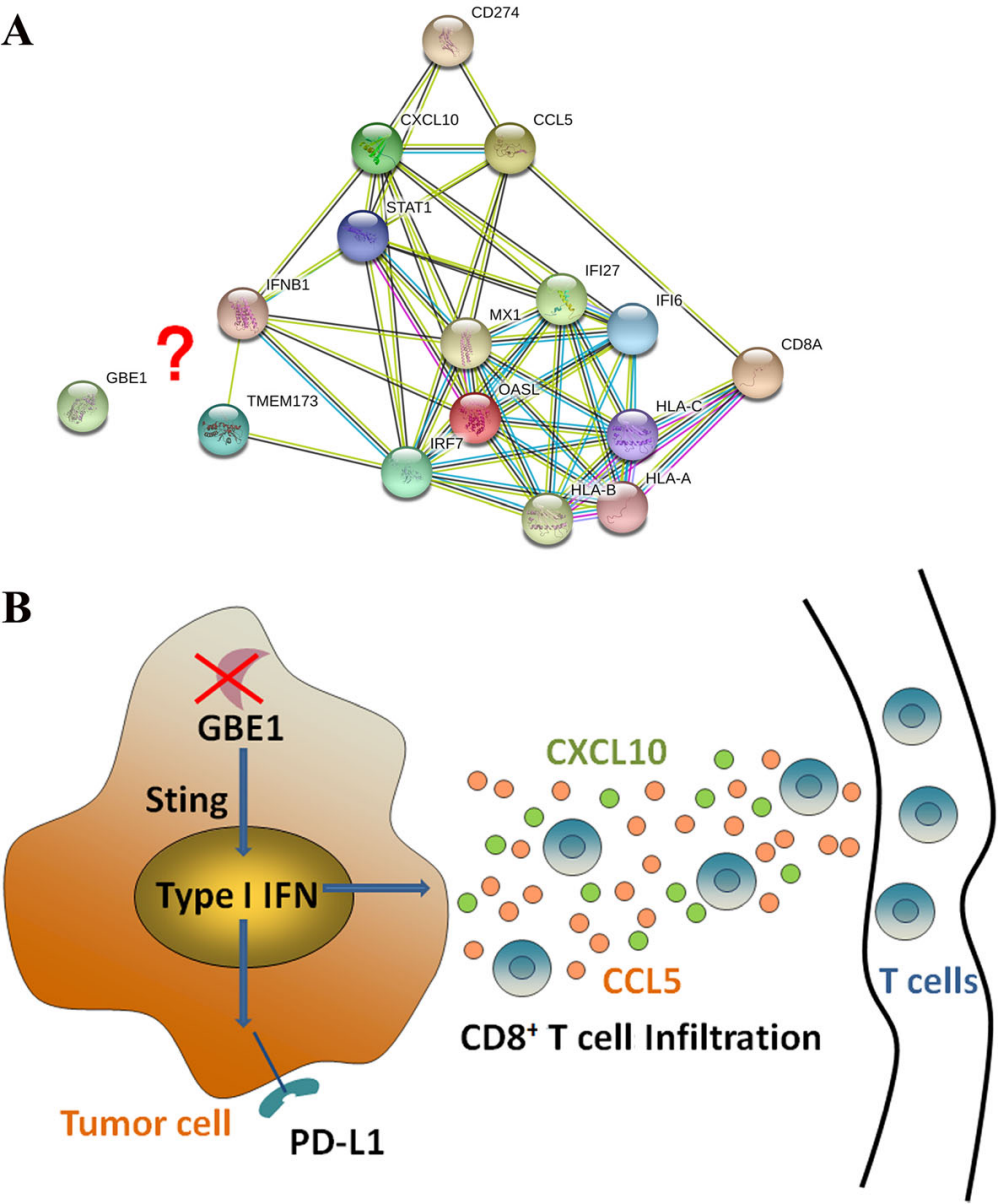
The summary and schematic diagram of this study**. a** STRING network analysis of GBE1 with CD8, PD-L1, CCL5, CXCL10, TMEM173, and IFNα/β pathway in the database. **b** Graphical abstract of this study. GBE1 blockade induces IFN-I production via STING signaling pathway, accompanied by upregulation of PD-L1 in LUAD cells, which further enhances the secretions of CCL5 and CXCL10 to recruit CD8^+^ T lymphocytes in the tumor microenvironment. Hence, suggesting that GBE1 may be a promising target to facilitate tumor rejection through cancer immunotherapy in LUAD

STRING (https://string-db.org/) network analysis showed that the relationship between GBE1 and the immune-related molecules analyzed in this manuscript is rarely reported (Fig. 7a). Collectively, these results indicate that GBE1 blockade induces IFN-I production via STING signaling pathway, accompanied by upregulation of PD-L1 in LUAD cells, which further enhances the secretion of CCL5 and CXCL10 to recruit CD8^+^ T lymphocytes in the tumor microenvironment. Therefore, GBE1 may be a promising target for cancer immunotherapy to achieve tumor regression in LUAD (Fig. 7b).

## Discussion

GBE1 (80 kDa, 702 aa) catalyzes the transfer of α-1,4-linked glucose units from the outer ‘non-reducing’ end of a growing glycogen chain into an α-1,6 position of the same or neighboring chain. GBE1 is required for the globular and branched structure of glycogen, which is essential to increase its solubility by creating a hydrophilic surface and reduce the osmotic pressure within cells [29, 30]. Our RNA-seq analysis indicated that the majority of changes driven by cytokine-cytokine receptor interactions and IFN-I signaling pathway occur before and after GBE1 knockdown. Reduced expression of GBE1 not only affects glucose metabolism pathways but also has a broader effect on the tumor microenvironment, ultimately resulting in reduced LUAD cell growth in vitro and in vivo. This study explored the mechanistic details of glycogen metabolism by GBE1 in tumor cells for its critical role in tumor growth and enhancing malignancy under hypoxia. With a growing interest in understanding how metabolic regulation controls the functional effector responses of immune cells, our study outlines an intricate and novel layer of complexity explaining how a metabolic pathway operates at a subcellular level, which may be exploited in cell-based therapeutic applications in the future.

STING, also known as TMEM173, MITA, ERIS, and MPYS, is an adapter that is activated by cyclic dinucleotides generated by cyclic GMP-AMP synthase (cGAS), which in turn is directly activated by cytosolic DNA. Activated STING forms aggregates in a perinuclear region and leads to the activation of tank-binding kinase 1 (TBK1), which in turn phosphorylates interferon regulatory factor 3 (IRF3) that directly contributes to IFN-I gene transcription [31–33]. The activation of the STING pathway is linked to the spontaneous generation of a T cell inflamed tumor microenvironment. Moreover, the strategies that activate or mimic the output of the host STING pathway should have immunotherapeutic potential in the clinic. A recent study has linked the activation of STING and production of inflammatory cytokines to brain metastasis and chemoresistance [34]. These studies indicate that in some conditions STING activation might facilitate inflammation-induced carcinogenesis; thus, an appropriate balance in STING pathway activation may be required for optimal anti-tumor effects [35, 36]. In the present study, we showed that blockade of GBE1 could promote anti-tumor immunity via activation of the IFN-I pathway through STING signaling.

The IFN-I family consists of genes encoding multiple IFN-α subtypes, one IFN-β, as well as the less-studied IFN-ε, −κ, −τ, and -ω subtypes, and the role of IFN-Is is critical in the early stages of anti-tumor immune response. The association between the IFN-I gene signature and T cell infiltration in human cancers as well as mouse tumor models have allowed a focus on innate signaling pathways capable of inducing IFN-I [37, 38]. A gene expression profile including an IFN-I signature showed a positive prognostic value in breast cancer [39–41], suggesting that IFN-I production might be integrally involved with adaptive T cell responses against tumor growth. In various cancers, there is a positive correlation between the expression of IFN-I and the presence of T cell markers in the tumor microenvironment. Production of IFN-β was detected in response to tumor challenge in tumor-draining lymph nodes, predominantly by CD11c^+^ cells consistent with DCs as a major source. Other cells in the tumor microenvironment may contribute to the production of IFN-Is, including tumor endothelial cells [42]. In our study, we also showed that IFN-I signature genes were closely associated with T cell infiltration including T cell marker CD8 and chemokines CCL5 and CXCL10 recruiting T cells to tumor sites.

We found a significant positive correlation between tumor PD-L1 expression and CD8^+^ T cell count. Tumor tissues from patients with triple-negative breast cancer have an increased number of TILs accompanied by increased PD-L1 level [43]. These observations suggested that these immunosuppressive mechanisms might not be associated with the tumor cells themselves but alternatively represent immune-intrinsic negative feedback processes that follow the recruitment of activated CD8^+^ T cells. A mechanistic study in mice confirmed that CD8^+^ T cells were required for the upregulation of PD-L1 within the tumor microenvironment [44]. Clinical responses observing immunotherapeutic interventions are strongly associated with the baseline presence of a CD8^+^ T cell infiltration [45, 46]. Gene expression profiling of the tumor microenvironment has revealed the presence of T cell transcripts, chemokines, and an IFN-I gene signature in a majority subset of human cancers [45, 47, 48]. Correlations observed between tumor PD-L1 expression and CD8^+^ T cell count indicate that tumor PD-L1 expression is related to host-tumor immunity, and thus, reflects the patient outcome. It may be possible that PD-L1 on tumor cells induces functional impairment of tumor-specific T cells without reducing their number as reported for antiviral T cells [49, 50]. However, the reduction of CD8^+^ T cells may not be the only mechanism by which PD-L1 promotes tumor immune escape. These results are in contrast with a previous report on ovarian cancer, in which an inverse correlation was observed between CD8^+^ T cell count and PD-L1 expression [51]. This discrepancy may reflect the distinct relationship between PD-L1 and CD8^+^ T cells depending on the organ or cancer type.

Blocking the PD-1/PD-L1 axis is recognized as an attractive target for cancer immunotherapy [52]. However, PD-1/PD-L1 blockade therapy is only successful in a small minority of patients with PD-L1 positive tumors that are infiltrated with cytotoxic lymphocytes. Such tumors are referred to as “hot” or “inflamed” tumors, which are present in only 10–20% of patients across all tumor types [53–56]. Therefore, the development of innovative treatment strategies that can increase PD-L1 expression and work in combination with immune cells to promote tumor infiltration is required. This would improve the overall success of PD-1/PD-L1 blockade therapy and benefit a greater number of patients. Our experimental results confirm the notion that upregulation of the PD-1/PD-L1 signaling pathway in tumor tissue, is a consequence of IFN-I activation and invasion by T cells, and predicts therapeutic benefit from therapeutic PD-1/PD-L1 blockade alone. Therefore, we propose that the expression of PD-L1 and IFN-I-responsive genes in tumor tissues could serve as sensitive biomarkers for patient stratification in clinical trials investigating PD-1/PD-L1 antibody-containing regimens. Since PD-L1 expression is generally thought to suppress the activity of immune cells, this hypothesis is in contrast with the currently established knowledge regarding PD-L1. However, the success of PD-1/PD-L1 checkpoint blockade as measured by the improvement in OS strongly correlates with PD-L1 expression on tumors [55].

Within the tumor microenvironment, chemokines play crucial roles in T lymphocyte recruitment [57, 58]. Apart from the effects they have on tumor cells, chemokines may also affect the tumor microenvironment [57, 59], particularly on the subtypes and frequencies of infiltrated T lymphocytes [47, 60]. CD8^+^ T lymphocytes infiltrated into specific sites are correlated with special chemokines [6, 7, 61]. Our data showed that chemokines CCL5 and CXCL10 promoted CD8^+^ T lymphocyte infiltration in LUAD, which was mediated by blockade of GBE1. Although CCL5 promotes the recruitment of regulatory T cells (Tregs) into tumor site [62], we did not find any significant differences in Treg infiltration in the xenograft tissues between the four groups in the in vivo experiment.

It has been shown that tumor-intrinsic active β-catenin signaling results in decreased CCL4 production, which further induces T-cell exclusion and resistance to anti-PD-L1/anti-CTLA-4 monoclonal antibody therapy [63]. Cancer-FOXP3 directly activates CCL5 to recruit FOXP3^+^ Treg cells in pancreatic ductal adenocarcinoma [62]. Our study also identified the function of cytokines/chemokines in cancer and immune cell interaction.

Glycogen metabolism has been previously implicated in myeloid cells of the immune system [64–68]. The role of glycogen metabolism, particularly GBE1, in immune effector accumulation of CD8^+^ T cells has not been previously elucidated. Here, we show a definitive role of GBE1 in the infiltration of T lymphocytes into tumor sites. We further demonstrate that depletion of GBE1 can upregulate CCL5 and CXCL10 expression through STING signaling to activate IFN-I pathway, potentiate T cell infiltration, and cause induced expression of PD-L1 on tumor cells simultaneously. Therefore, the results of the present study and the hypotheses derived from it suggest a potential anti-metabolic therapy for LUAD in combination with immune checkpoint blockade.

## Additional files

Additional file 1: **Figure S1.** CCL5 and CXCL10 expression in LUAD cells with GBE1 overexpression. **(A)** Western blotting analysis and **(B)** the statistical analysis confirms GBE1 overexpression in A549 cells compared to negative control cells. **(C)** Real-time PCR and **(D)** ELISA analysis of CCL5 and CXCL10 expression in A549 cells with GBE1 overexpression compared to control. Data are represented as means ± SD. *** = *P* < 0.001. (TIF 865 kb)

Additional file 2: **Figure S2.** Treg infiltration in the xenografts**.** The expression of FOXP3 in the xenografts was analyzed by IHC and one representative micrograph is shown (200 ×). The results are presented as a histogram. Data are represented as means ± SD. (TIF 5040 kb)

## Abbreviations

cGAS: GMP-AMP synthase
CTLs: cytotoxic T lymphocyte
GBE1: glycogen branching enzyme
GFP: green fluorescent protein
GO: Gene Ontology
IFN-I: type I interferon
IRF3: interferon regulatory factor 3
LUAD: lung adenocarcinoma
PD-1: programmed cell death protein-1
PD-L1: Programmed death ligand-1
poly(I:C): polyinosinic:polycytidylic acid
RNA-seq: RNA-sequencing
RSEM: RNA-seq by Expectation Maximization
shRNA: short hairpin RNA
siRNAs: small interfering RNAs
TBK1: tank-binding kinase 1
TCGA: The Cancer Genome Atlas
TILs: tumor infiltrating lymphocytes
