# Supplementary figures and images for: Lung adenocarcinoma-intrinsic GBE1 signaling inhibits anti-tumor immunity
Source: Mol Cancer. 2019 Jun 20;18:108. doi: 10.1186/s12943-019-1027-x (PMC6585057; doi:10.1186/s12943-019-1027-x)

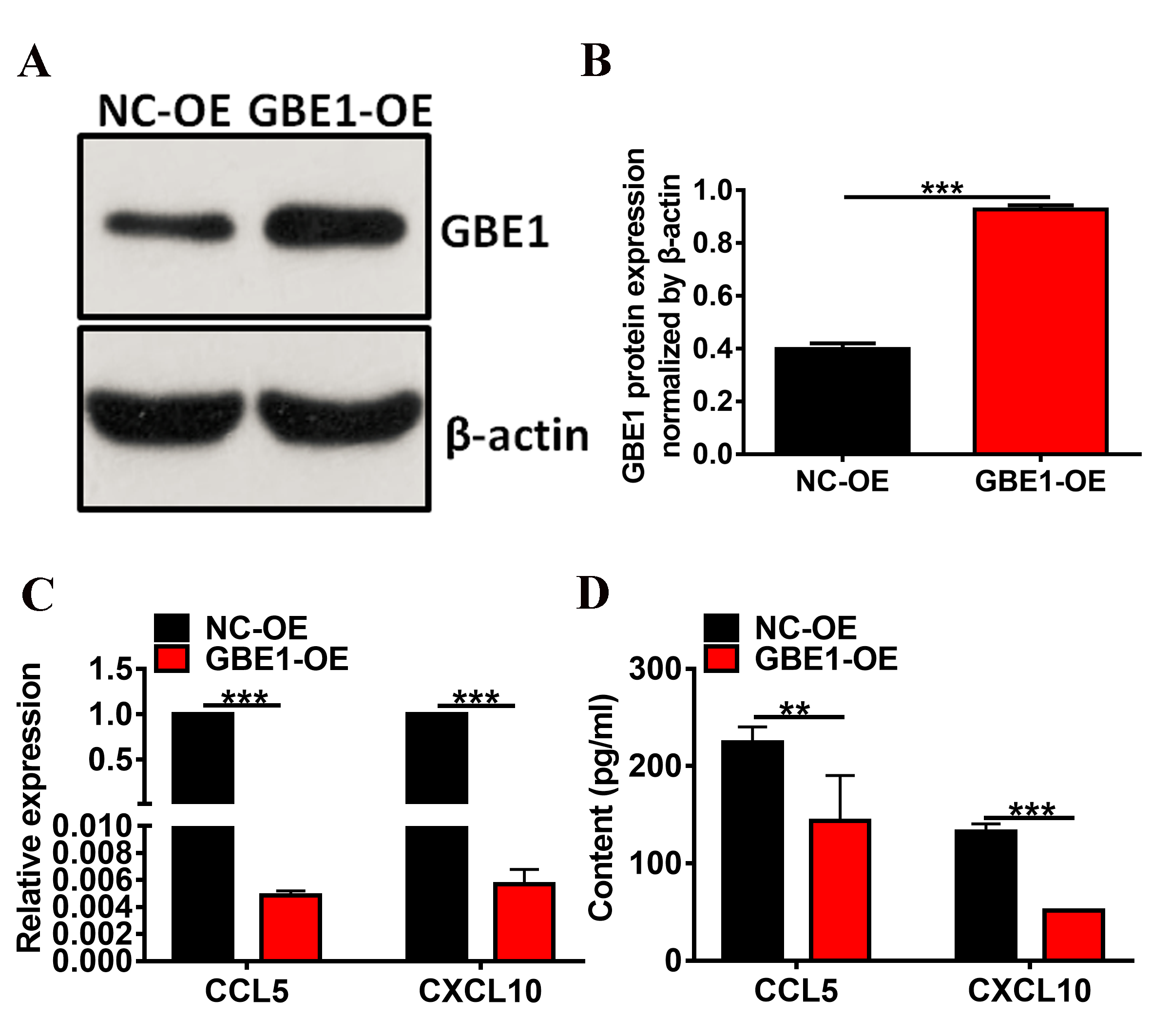

Supplement: Supplementary file 1 — Figure S1. CCL5 and CXCL10 expression in LUAD cells with GBE1 overexpression. (A) Western blotting analysis and (B) the statistical analysis confirms GBE1 overexpression in A549 cells compared to negative control cells. (C) Real-time PCR and (D) ELISA analysis of CCL5 and CXCL10 expression in A549 cells with GBE1 overexpression compared to control. Data are represented as means ± SD. *** = P < 0.001. (TIF 865 kb) [file 12943_2019_1027_MOESM1_ESM.tif]

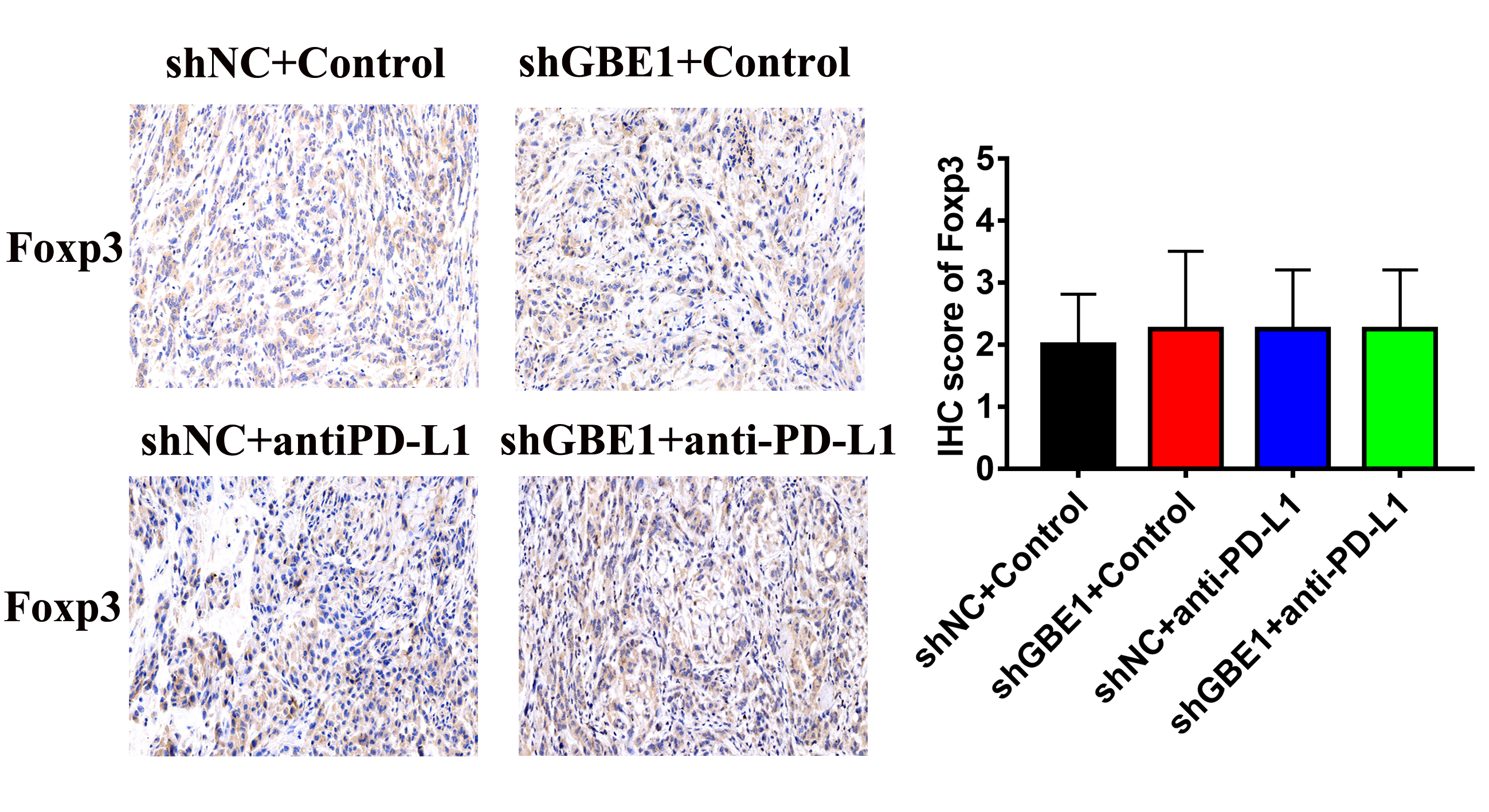

Supplement: Supplementary file 2 — Figure S2. Treg infiltration in the xenografts. The expression of FOXP3 in the xenografts was analyzed by IHC and one representative micrograph is shown (200 ×). The results are presented as a histogram. Data are represented as means ± SD. (TIF 5040 kb) [file 12943_2019_1027_MOESM2_ESM.tif]
